# Effect of estrous cycle phases on gene expression in bovine oviduct epithelial cells

**DOI:** 10.14202/vetworld.2022.1665-1675

**Published:** 2022-07-14

**Authors:** Ricaurte Lopera-Vásquez, Fabián Uribe-García, Iang Rondón-Barragán

**Affiliations:** 1 Impronta Research Group, Faculty of Veterinary Medicine and Zootechnics. Universidad Cooperativa de Colombia, Ibagué-Tolima, Colombia; 2 Research Group in Immunobiology and Pathogenesis, Laboratory of Immunology and Molecular Biology, Faculty of Veterinary Medicine and Zootechnics, Universidad del Tolima, Santa Helena Highs, Postal Code 730006299, Ibagué-Tolima, Colombia

**Keywords:** bovine oviduct epithelial cells, estrus phase, follicular, gene expression, luteal, oviduct

## Abstract

**Background and Aim:**

The oviduct environment is of particular importance because it is the site of fertilization and early embryo development. The oviduct, as a component of the reproductive system, responds to ovarian hormone (estradiol [E2] and progesterone [P4]) stimuli depending on the estrous cycle phase. This study aimed to elucidate the effect of estrous cycle phases (follicular and early and late luteal phases) on gene expression patterns in bovine oviduct epithelial cells (BOECs).

**Materials and Methods:**

Oviducts were obtained from healthy slaughterhouse animals, corresponding to ipsilateral ovaries with dominant follicles or corpus luteum during early and late luteal phases. BOECs were recovered from the isthmus (IST) and ampulla (AMP), and the expression patterns of genes related to cytokinesis and mitosis mechanisms (rho-associated coiled-coil containing protein kinase and cellular communication network factor 2 [*CCN2*]), growth factors (insulin-like growth factor-binding protein 3, epidermal growth factor receptor [*EGFR*], vascular endothelial growth factor A, and *EGFR*), antioxidant mechanisms (glutathione peroxidase 4 [*GPX4*]), apoptosis (B-cell lymphoma 2), complement component (*C*3), energy metabolism (aldose reductase gene family 1-member b1 [*AKRIB1*] and solute carrier family 2), hormone receptors (estrogen receptor 1 and luteinizing hormone/choriogonadotropin receptor), and specific glycoproteins (oviductal glycoprotein 1) were analyzed.

**Results:**

High P4 levels (late luteal phase) affected the expression of important genes related to antioxidant mechanisms (*GPX4*), energy metabolism (*AKRIB1*), growth factors (*IGBP3* and *EGFR*), and cell growth regulation (*CCN2*) in the AMP. Low P4 levels (early luteal phase) affected the expression of *AKR1B1*, *IGBP3*, and *CCN2*. In addition, estrogen likely had an effect on *OVPGP* expression in the cattle oviduct.

**Conclusion:**

Differential gene expression patterns of BOECs in the AMP during the luteal phase (antioxidant mechanisms, energy metabolism, growth factors, and immunological regulators) and in the IST during the follicular phase (glycoproteins) may influence their renewal and population proportions, modulating the oviduct environment as well as gamete and embryo physiology.

## Introduction

The bovine oviduct is the part of the female reproductive system where fertilization and early embryo development occur, providing an optimum environment for final oocyte maturation, sperm capacitation, sperm storage, and fertilization as well as gamete and embryo transport [1–3]. The oviduct environment is primarily regulated by oviductal epithelial cell (OEC) secretion and plasma components [4]. OECs are crucial for gamete transport, maintenance, preparation, and early embryo development [5]. OECs can be ciliated or secretory (non-ciliated), and they are distributed in the oviduct ampulla (AMP) and isthmus (IST) [6], with the infundibulum and AMP containing the largest proportion of ciliated OECs [7].

Physiologically, hormones in the circulation reach OECs from the basolateral surface and regulate various cell functions [8]. During the estrous cycle, the oviduct is affected by ovarian sex hormones, mainly estrogens and progesterone, which result in morphological and biochemical changes for maintaining an appropriate environment for gamete and embryonic development [9]. The morphological development and ratio of ciliated and secretory OECs are completed during the estrous cycle. During the luteal phase, goat OEC cell height decreases, mainly in the ciliated cells (CCs) of the entire oviduct [10]. The number of CCs increases during the late luteal phase in the infundibulum and AMP and decreases as it approaches the IST [11]. In particular, protein synthesis in the oviduct peaks during the follicular phase due to estrogens [12], including the expression of oviductal glycoprotein 1 (*OVGP1*) and progesterone receptor [13].

The environment of the bovine oviduct has been studied *in vitro* and *in vivo*, enabling the characterization of secreted proteins and their roles in the oviduct environment [14]. Regulatory mechanisms for genomic pathways [15] affect capacitation, fertilization capacity, and early embryo development and competence. Schmaltz-Panneau *et al*. [16] found variations in OEC gene transcription in developing embryos. Therefore, studying the oviduct environment is crucial for understanding the underlying regulatory mechanisms that influence embryo development [17].

The use of bovine oviduct epithelial cells (BOECs) provides significant advances in the *in vitro* culture (IVC) systems of bovine embryos [18], with implications for embryo development and competence [19]. These advancements enable the development of further technologies, particularly those related to early embryo development and IVC, and establish the BOEC coculture as an appropriate model for studying the pathways for embryo-maternal communication in bovines [20].

Changes in the activity or abundance of several molecules during the estrous cycle may be crucial for the oviduct environment [21]. These molecules positively affect sperm motility, capacitation, and gamete interaction [22]. Transcriptomic studies detected a relationship between estrous cycle-dependent changes in gene expression levels and morphological changes in BOECs [23, 24]. The gene transcript upregulated in the luteal phase (under progesterone [P4] influence) is associated with the regulation of cell proliferation. In contrast, those upregulated in the follicular phase (under estradiol [E2] influence) are involved in protein modulation [25].

This study aimed to elucidate the differential expression of various BOEC genes during the estrous cycle.

## Materials and Methods

### Ethical approval

No ethical approval was required for this study because oviduct samples were recovered from bovine females from the slaughterhouse.

### Study period and location

The study was carried out from January to August 2021. Molecular experiments were done in the Laboratory of Immunology and Molecular Biology – LIBM of the University of Tolima.

### BOEC isolation and preparation

Following slaughter, the female reproductive tract was recovered from healthy animals, and oviducts (ipsilateral) were collected based on ovarian morphology using the criteria described by Ireland *et al*. [26]. Oviducts were separated from connective tissue, ligated, dissected, and washed in phosphate-buffered saline (PBS) before being transported on ice to the laboratory in PBS with 2% penicillin-streptomycin solution (Sigma^®^, Germany). Before removing the closure in a laminar flow hood, the oviducts were briefly soaked in ethanol and rinsed in PBS. The oviduct was divided into two equal segments (AMP and IST), and each segment was gently squeezed in a stripping motion using a sterile glass slide. The oviduct content (yellowish paste) was washed twice with PBS by centrifugation at 300× g for 10 min [27] and then collected in a vial for RNA extraction. The ovarian structures identified for oviduct classification were as follows: (a) Dominant follicle at the follicular phase; (b) hemorrhagic corpus luteum at the early luteal phase; and (c) total corpus luteum at the mid-luteal phase of the estrous cycle [26].

### Experimental design

Gene expression analyses were performed in the oviduct AMP and IST during the early luteal phase on days 2–6 (hemorrhagic corpus luteum, early), the mid-luteal phase on days 6–17 (functional corpus luteum, late), and the follicular phase on days 17–21 (follicular).

### RNA extraction and cDNA synthesis

For each gene, a set of primers was designed using Geneious Prime v2022.1 (https://www.geneious.com/prime/), according to Kearse *et al*. [28] (Table-1). Total RNA was extracted using the TRIzol method (Thermo Fisher Scientific, USA Life Technologies; Cat# 15596018) and cDNA was synthesized using the SuperScript^®^ IV First-Strand Synthesis System (Thermo Fisher Scientific, USA). The relative expression of genes of interest was normalized using glyceraldehyde 3-phosphate dehydrogenase (*GAPDH*) as a reference gene and calculated using the 2^-∆∆Ct^ method [29]. Quantitative real-time polymerase chain reaction (qPCR) was performed using QuantStudio 3 (Thermo Fisher Scientific); the Luna Universal qPCR Master Mix (New England Biolabs, USA) was used. Melting curve analysis was performed to rule out gDNA contamination and non-specific primer annealing.

**Table 1:** Sequence of primers used for qPCR.

Gene name	Primer sequence (5´- 3´)	Amplicon size (bp)	GenBank accession no.
*ROCK2*			
F	GCCTTGGAAGAAACGGGGTA	191	NM_174452.2
R	AGCCTTGGGAATTGGGAAGG		
*GPX4*			
F	CAAGCCTGTTGCCTGTGTTC	173	NM_001346430.1
R	TATTCCCACAAGGCAGCCAG		
*IGFBP3*			
F	GTCGGAAGAAGACCACAGCA	178	NM_174556.1
R	CTGGGTGTCTGTGCTCTGAG		
*ESR1*			
F	AGGCAGAGAATTTCCCCAGC	175	NM_001001443.1
R	GCGAATGAATGGCCATCCAC		
*VEGFA*			
F	CAAACCTCACCAAAGCCAGC	186	NM_001316955.1
R	CGCGAGTCTGTGTTTTTGCA		
*OVGP1*			
F	GCTGTCCACGTTTTCCAACC	192	NM_001080216.1
R	GTGAGCTGGGCCTCATTCTT		
*C3*			
F	CACTGTTGACCACAAGCTGC	95	NM_001040469.2
R	AAGACTTGGAGTCCCGCTTG		
*EGFR*			
F	ACTCTGATGCTGGGAGGGAT	126	XM_002696890.5
R	CCTGTCCATCACCAACTCCC		
*LHCGR*			
F	GAGAAGATGCACAACGACGC	193	NM_174381.1
R	AAGTCAGTGTGGCATCCAGG		
*SLC2A1*			
F	TGGGAGGCATGATTGGTTCC	157	NM_174602.2
R	CGACCCAGGATCAGCATCTC		
*BCL2L2*			
F	CGTGTGAGGGTGACGTGTAA	155	NM_001076533.1
R	CACCCATGCAAACAGTGTGG		
*AKR1B1*			
F	TAATTTGGCCCGTGTCCCTC	163	NM_001012519.1
R	TTGCATGTTCCCCAGATCCC		
*CCN2*			
F	TCTTCGGAGTGTGCACAGAC	134	NM_174030.2
R	GTAATGGCAGGCACAGGTCT		
*GAPDH*			
F	GGGATGAGGCTCAGAGCAAGAGA	118	NM_173979.3
R	AGCTCGTTGTAGAAGGTGTGGTGCC		

qPCR=Quantitative real-time polymerase chain reaction

### Statistical analysis

Data were analyzed using descriptive statistics and normality tests. Gene expression levels were compared among early, late, and follicular phases of the estrous cycle in AMP and IST using a one-way analysis of variance with Tukey’s test as *post hoc*. Statistical analyses were performed using the GraphPad Prism software v8.0 for MacOS (Dotmatics; La Jolla, CA, USA). Differences with p < 0.05 were considered statistically significant.

## Results

**Figure-1:**
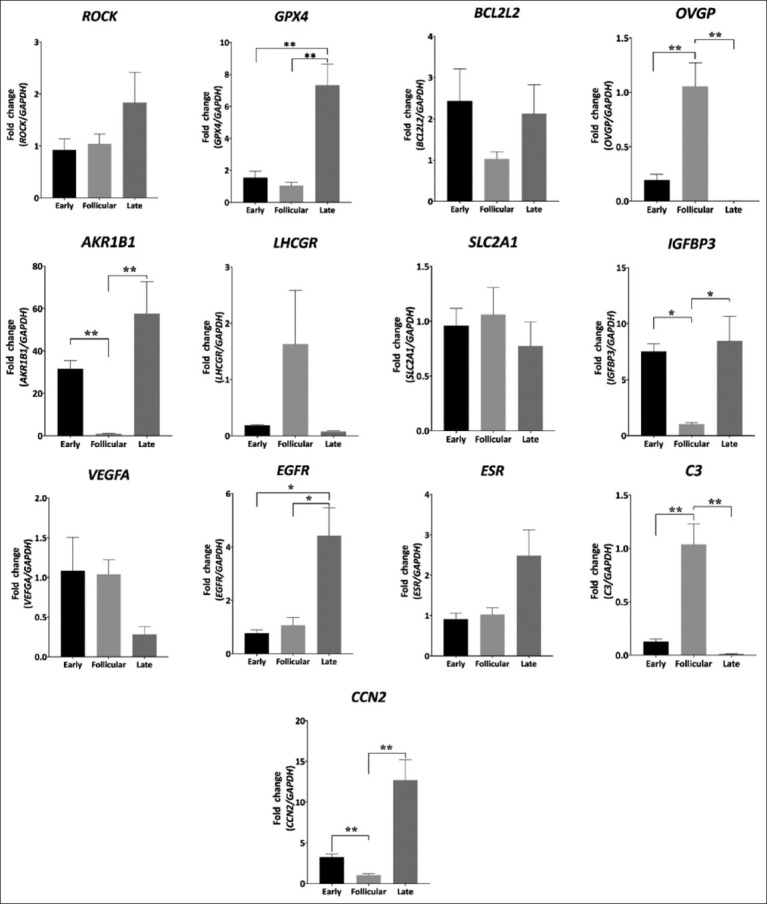
Gene expression patterns of different genes related to cytokinesis and mitosis mechanisms (*ROCK*, rho-associated coiled-coil containing protein kinase; *CCN*, Cellular communication network), growth factors (*IGFBP3*, insulin-like growth factor-binding protein 3; *EGFR*, epidermal growth factor receptor; *VEGFA*, vascular endothelial growth factor A), antioxidants mechanisms (*GPX4*, glutathione peroxidase 4), apoptosis (*BCL2*, B-cell lymphoma 2), complement component (*C3*), energetic metabolism (*AKRIB1*, aldose reductase gene family 1-member b1; *SLC2A1*, solute carrier family 2, facilitated glucose transporter member 1), hormones receptors (*ESR*, estrogen receptor; *LHCGR*, luteinizing hormone/choriogonadotropin receptor), and specific glycoproteins (*OVGP*, oviduct-specific glycoprotein) on oviduct ampulla, during different estrous cycle phases. Early: Early luteal phase, Late: Mid-luteal phase, Follicular: Follicular phase. Data are shown as mean ± SEM. *p < 0.05; **p< 0.01. SEM=Standard error of the mean.

**Figure-2:**
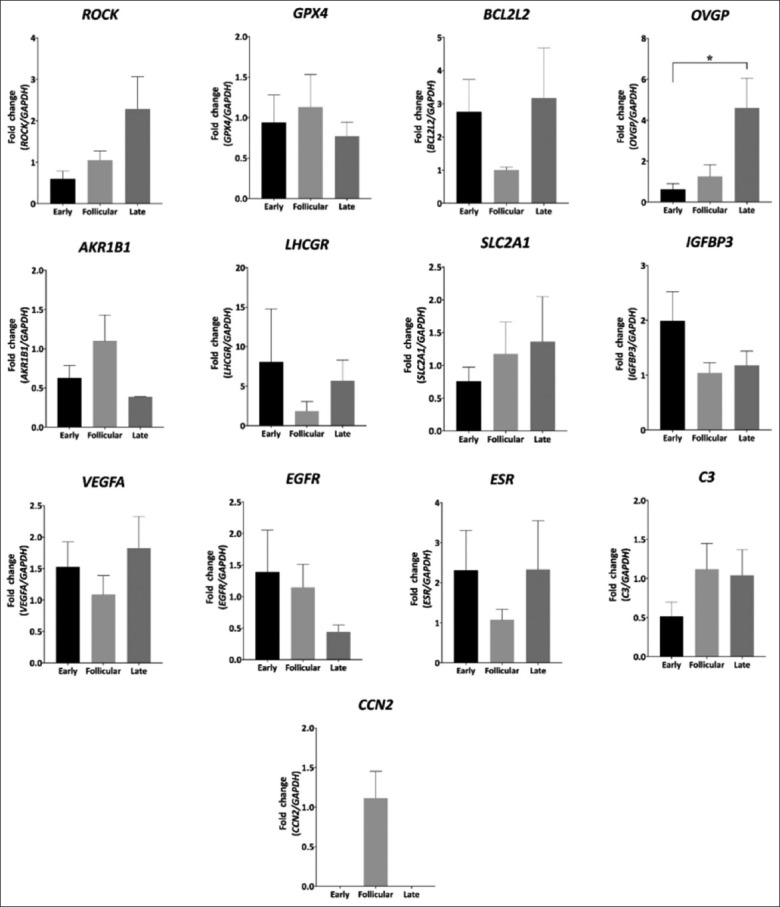
Gene expression patterns of different genes related to cytokinesis and mitosis mechanisms (*ROCK*, rho-associated coiled-coil containing protein kinase; *CCN*, Cellular communication network), growth factors (*IGFBP3*, insulin-like growth factor-binding protein 3; *EGFR*, epidermal growth factor receptor; *VEGFA*, vascular endothelial growth factor A), antioxidants mechanisms (*GPX4*, glutathione peroxidase 4), apoptosis (*BCL2*, B-cell lymphoma 2), complement component (*C3*), energetic metabolism (*AKRIB1*, aldose reductase gene family 1-member b1; *SLC2A1*, solute carrier family 2, facilitated glucose transporter member 1), hormones receptors (*ESR*, estrogen receptor; *LHCGR*, luteinizing hormone/choriogonadotropin receptor), and specific glycoproteins (*OVGP*, oviduct-specific glycoprotein) on oviduct isthmus, during different estrous cycle phases. Early: Early luteal phase, Late: Mid-luteal phase, Follicular: Follicular phase. Data are shown as mean ± SEM. *p < 0.05; **p < 0.01. SEM=Standard error of the mean.

The transcript levels of glutathione peroxidase 4 (*GPX4*), aldose reductase gene family 1-member b1 (*AKRIB1*), cellular communication network factor 2 (*CCN2*) were significantly higher (p < 0.01), same as insulin-like growth factor-binding protein 3 (*IGFBP3*), and epidermal growth factor receptor (*EGFR*) levels which were higher in the AMP (p < 0.05) in the late luteal phase (late) than in the follicular phase (follicular), similar to transcript levels patterns of *GPX4* and *EGFR* that were higher in late compared to the early luteal phase (early) (p < 0.05). Oviductal glycoprotein 1 (*OVGP1*) and complement C3 (C3) were significantly higher (p < 0.01) in the follicular phase than in the late and early phases. The early phase showed a higher *AKR1B1* (p < 0.05), and significantly higher *IGFBP3*, and *CCN2* (p < 0.01) expression than the follicular phase, whereas no significant differences in Rho-associated coiled-coil containing protein kinase (*ROCK*), B-cell lymphoma 2 like 2 (*BCL2L2*), luteinizing hormone/choriogonadotropin receptor (*LHCGR*), solute carrier family 2 (*SLC2A1*), vascular endothelial growth factor A (*VEGFA*), and estrogen receptor 1 (*ESR1*) expression (Figure-1) were observed. In the IST, *OVGP1* expression was higher in the late phase than in the early and follicular phases (p < 0.05). However, *ROCK*, *GPX4*, *BCL2L2*, *AKRIB1*, *LHCGR*, *SLC2A1*, *IGFBP3*, *VEGFA*, *EGFR*, *C3*, and *CCN2* expression did not differ significantly between phases (Figure-2).

## Discussion

The oviduct is a dynamic organ that is anatomically divided into three different parts: The infundibulum, the AMP, and the IST, and it has the potential to provide a suitable microenvironment for gametes and embryo development [30]. The previous studies have demonstrated that genes are differentially expressed between oviductal regions and that it is related to the estrous cycle [23, 30, 31], including variations in oviduct epithelial transcription [32]. Our findings indicate that gene expression in BOECs differs between the AMP and IST as well as among the follicular, early, and late estrous phases. This differential gene expression may be related to BOEC functional differentiation in each oviduct region. Thus, understanding specific regulatory mechanisms that are affected under the influence of E2 and P4 may help expand the knowledge of oviductal physiology, including signaling pathways and their modulation in the oviductal environment, to enhance the *in vitro* embryo culture environment.

The oviduct’s function is dependent on its OECs, which are classified into CCs and secretory cells (SCs). SCs have secretory activity and may be larger than CCs. They exude nutrients into the oviduct lumen for gamete and embryo development and form cellular protrusions that increase in size and number as the estrous cycle progresses [33–35]. In particular, when oocytes are in the AMP after ovulation, SCs exhibit morphological changes indicative of increased activity [36], implying a link between SC morphology and endocrine levels during the estrous cycle [37]. On the other hand, CCs have hair-like projections (cilia) that generate a ciliary beating and sweep mucus and other debris off the epithelium and oviduct lumen. Changes in cilia orientation and beat frequency can modify oviduct fluid flow, which could affect gamete and embryo transport [38].

Ovarian steroids (E2 and P4) regulate oviductal function, particularly gamete and early embryo transport, by modulating SC and CC physiology [39]. E2 increases SC proliferation, secretory activity, and CC transport velocity, whereas P4 decreases SC secretory activity and CC transport velocity while initiating a process of self-renewal of the BOECs for the subsequent estrous cycle. This BOEC renewal, which is required for proper maternal receptivity, is regulated by proliferation, differentiation, and apoptosis [40].

This study analyzed ROCK and CCN2 gene transcripts, which are related to OEC renewal. Rho-associated coiled-coil containing protein kinase (*ROCK*) regulates cell division, cell-matrix adhesions, and the activation of the c-Fos serum response element [41]. Our findings indicate that the estrous phase and oviduct region have no effect on *ROCK* expression patterns, implying the absence of Rho-dependent kinase mechanisms during different estrous phases. On the other hand, *CCN2* mRNA levels were upregulated in the AMP (late-early), with no differential expression in the IST, which may be related to P4 secretion. Cyclin B1 (*CCN*) is a regulatory protein that is involved in both the oocyte cell cycle and embryonic development. It assembles a complex with p34 (cyclin-dependent kinase 1) to form mitosis-promoting factor, which is required for the proper control of the G2/M transition phase of the cell cycle [42]. Genistein, which induces cell cycle arrest, downregulates *CCN* and *BCL-2/BAX* expression in BOEC primary cultures, resulting in reduced cellular proliferation and migration [43]. In mice, maternal factors and ovarian steroids regulate *CCN2* (*CTGF*) and transforming growth factor-beta 1 expression in the uterus [44]. The differential expression of both genes reflects the dynamics of cytokinetic processes during the estrous cycle phases in oviduct regions.

Growth factors play a pivotal role in the oviduct microenvironment, modulating cell proliferation, wound healing, and cellular differentiation [45]. In this study, *IGFBP3*, *EGFR*, and *VEGFA* gene expression were analyzed. *IGFBP3* is an IGF-binding protein [46]; in BOECs expressing *IGFBP3*, particularly in the IST, it may help create a synchronized IGF-I and IGF-II gradient along the oviduct as the embryo passes through it [47, 48]. BOEC *IGFBP3 sec*retion increases in response to early embryo development in a conditioned medium [49]. Our findings suggest that this growth factor receptor in the AMP may be responsive to P4, exhibiting increased expression during the early and late luteal phases, corresponding to the early embryo development till day 4.

These patterns were similar to those of the *EGFR*, where P4 (late-early) had an effect on the AMP. *EGFR* is a transmembrane glycoprotein of the protein kinase superfamily [50] that binds to EGF, promoting receptor dimerization and tyrosine autophosphorylation, which promotes cell proliferation [51]. It may also be involved in the regulation of the bovine oviductal microenvironment [52] by modulating OEC renewal and oviduct lumen characteristics by controlling apoptotic gene expression (*BAX* and *BCL-2*) [53]. The mid-luteal stage exhibits enhanced EGF secretion and *EGFR* mRNA expression in the bovine endometrium, which may regulate uterine function [54].

Angiogenesis plays an important role in cyclical mechanisms during the estrous cycle, such as follicular growth and corpus luteum formation, which are dependent on the development of a dense capillary net [55], and transudation from plasma contributes to the defining characteristics of the luminal microenvironment in cattle, such as uterine and oviductal fluid [56]. *VEGFA* is a member of the platelet-derived VEGF family that encodes a heparin-binding protein, which promotes vascular endothelial cell proliferation and migration [57]. Our findings indicate that the estrous phase and oviduct region have no effect on the expression patterns of this gene. However, a previous study in heifers demonstrated that low follicle count downregulated *VEGFA* expression in ipsilateral oviducts compared to the contralateral IST [58]. VEGF and its receptors, flk-1 and flt-1a, are secreted in the bovine oviduct [59], where they have autocrine and paracrine effects on BOECs, influencing physiological functions through permeability modifications. VEGFs, which are angiogenic in nature, are associated with vascular support in the oviduct, being directly associated with OEC support and oviductal fluid perfusion.

The redox state of the bovine oviduct is pivotal for gamete physiology and embryo survival, and antioxidant expression has been reported [60, 61]. *GPX4* catalyzes the reduction of hydrogen peroxide and organic and lipid hydroperoxides, thereby protecting cells from oxidative stress [62]. *GPX4* is also essential for sperm development; consequently, it is classified as a “moonlighting” protein based on its ability to function as both a peroxidase and a structural protein in mature spermatozoa [63]. *GPX4* transcript levels decreased in BOEC cultured embryos [49] but increased in the IST ipsilateral to the dominant follicle during the follicular phase and in the post-ovulatory period [64]. Our findings suggest that P4 levels (late) have an effect on *GPX4* expression in the AMP. Previously, a significantly increased transcript abundance of *GPX4* in ipsilateral BOECs was observed in the early luteal phase [27], which is consistent with our findings. On the other hand, apoptosis mechanisms are well known in gametes and early embryos and are directly linked to their viability. Therefore, the presence of these factors in the oviduct environment should be investigated. An apoptosis regulator, B-cell lymphoma 2 (*BCL2*) control outer mitochondrial membrane permeabilization (MOMP) protein that regulates apoptosis mitochondrial pathway [65]. In contrast to the findings of *GPX4*, the estrous phase and oviduct region had no effect on *BCL2* expression patterns in our study. Recently, researchers found that increased *BCL2* and *BAX* mRNA expression in BOEC c onditioned media and exosomes influence apoptosis in embryos [5]. In other mammals, apoptosis levels are P4 dependent (caudal oviduct and uterotubal junction) and can affect spermatozoa bound to OECs as well as the fertilization process [66].

The C3a peptide, also known as C3a anaphylatoxin, is a complement component that regulates inflammation and has antimicrobial effects [67]. Complement proteins have been suggested to mediate functional homeostasis, which has been linked to physiological aspects of cattle oviduct and fertility [56], sperm-oocyte interaction [68], and embryo development [69]. In rodents, C3 is secreted by OECs and increases trophectoderm development, blastocyst size, and hatching rates [70, 71]. Our findings suggest that C3 transcription levels in the oviduct AMP may be influenced by E2 levels.

Glucose is an essential energy source for mammalian pre-implantation embryo development, and oviduct glucose and lactate concentrations have been linked to oviduct and epithelial differential regulation [72]. Glucose and lactate levels are important to gamete and early embryo processes, and multiple genes are involved in regulatory mechanisms. The *AKRIB1* is a key enzyme that catalyzes the reduction of several aldehydes as well as steroid metabolism [73], including P4 (for implantation) [74] and prostaglandin F2α synthesis (luteolysis and completing pregnancy). The increased *AKRIB1* expression in our study indicates a possible effect of P4 on the AMP during late and early phases, which was not evident on the IST, and can influence the oviductal environment, including early embryos in transit during the first stages of development. In bovines, *AKR1B1* upregulation in blastocyst biopsies influences the pregnancy absence and endometrial resorption during luteolysis, and it has been related to early embryo pro-apoptotic pathways [75]. Similarly, solute carrier family 2 (*SLC2A1*), facilitated glucose transporter member 1 (SLC2A1), or GLUT1, is a major glucose transporter in the mammalian blood–brain barrier, mammary gland, kidney, muscle [76], fetus [77], and ovary [78], where it is responsible for maintaining basal glucose [79]. In the present study, *SLC2A*1 mRNA level did not vary between the estrous phase and the oviduct region, contrasting with previous studies where *SLC2*A showed differential expression in murine early embryos and laying hen oviducts based on glucose metabolism [80, 81].

E2 has an effect on several regions of the reproductive tract, including the oviduct, and its receptors are essential for sexual development and reproductive activity [82]. E2 concentrations during the estrous phase suggest that a large amount of E2 is delivered to the oviduct [83]. Estrogen receptor ( *ESR*) is a ligand-activated transcription factor composed of several domains essential for E2 hormone-binding, DNA-binding, and transcription activation [84]. In particular, *ESR1* is highly expressed in the IST during the follicular phase [85]. In mice, disrupting the E2 signal in OECs causes defective embryo transport, implying that the E2 signal through *ESR1* is necessary for embryo transport in the oviduct. In humans, increased pre-ovulatory estradiol levels act through epithelial ERα, suppressing protease-mediated aspects of innate immunity in the oviduct, influencing the mucosal immunological barrier to support fertilization and embryo development [86]. However, the *ESR* mRNA levels in BOECs in our study were comparable between estrous phases in both oviduct regions, suggesting that receptor expression in BOECs is stable regardless of the estrous cycle phase.

The LHCGR belongs to the G-protein coupled receptor 1 family and its function is regulated by G proteins that activate adenylate cyclase [87]. Alterations in this gene influence the appearance of secondary sexual characteristics in males, such as familial male early puberty (testotoxicosis), secondary hypogonadism, Leydig cell adenoma (early puberty), and hypoplasia (male pseudohermaphroditism). However, BOEC luteinizing hormone: Human chorionic gonadotropin (LH: hCG) under exogenous LH: hCG promotes *OGP* expression by increasing transcript stability [88]. *LHCGR* expression levels on BOECs are reported for the first time in this study, and it is suggested that the estrous phase and oviduct region have no impact on its expression patterns.

Oviduct-specific glycoprotein (*OVGP*) is related to the mucin and glycosyl hydrolase 18 gene families. *OVGP* expression and protein secretion occur during final follicular development through cleavage-stage embryo development, suggesting that *OVGP* expression is E2 dependent. *OVGP* is secreted from SCs and associates with ovulated oocytes, blastomeres, and spermatozoan acrosome regions. *OVGP1* mRNA expression and its relationship with cell *in vivo* behavior constitute a marker for functionality [22], whereas it indicates OEC physiological phenotype under *in vitro* conditions [89]. In our study, *OVGP* expression was upregulated in the oviduct AMP and IST in response to E2 and P4. The effect of E2 on *OVGP* expression is comparable to an *in vivo* study finding, in which BOECs showed increased *OVGP1* mRNA expression in response to E2 [89], although the effect of P4 on *OVGP* expression was not described. However, due to the lack of significant variations in *ESR* expression, a clear association between this type of interaction and *OVGP* expression cannot be established.

Understanding the physiological events of the female reproductive tract necessitates an understanding of the expression patterns of functions that elucidate the dynamics of the oviductal environment during the estrous cycle, gamete transport, and early embryo development.

## Conclusion

The luteal phase (early and late) of the estrous cycle affected gene expression patterns, which were reflected in antioxidant mechanisms (*GPX4*), energy metabolism (*AKRIB1*), growth factors (*IGFBP3*, *EGFR*), and cell adhesion - communication ( *CCN2*) in the oviductal AMP, while the follicular phase influenced the gene expression of an essential glycoprotein, *OVGP*, in the oviductal IST. The dynamics of specific mechanisms in OECs, both in the AMP and IST, influence oviductal epithelial renewal and population proportions, which further modify oviductal lumen conditions, as well as oviductal fluid secretions and volume, which influence gamete and embryo physiology during transit.

## Authors’ Contributions

RL, IR, and FU: Conceived and designed the study. RL, IR, and FU: Performed the experiments. IR, RL, and FU: Analyzed the data. RL and IR: Drafted the manuscript. All authors have read and approved the final manuscript.
